# Collisionless cooling of perpendicular electron temperature in the thermal quench of a magnetized plasma

**DOI:** 10.1038/s41598-024-73968-7

**Published:** 2024-10-08

**Authors:** Yanzeng Zhang, Jun Li, Xian-Zhu Tang

**Affiliations:** 1 Theoretical Division, Los Alamos National Laboratory, Los Alamos, New Mexico 87545 USA; 2 School of Nuclear Science and Technology, University of Science and Technology of China, Hefei, 230026 Anhui People’s Republic of China

**Keywords:** Plasma physics, Astrophysical plasmas, Magnetically confined plasmas

## Abstract

Thermal quench of a nearly collisionless plasma against an isolated cooling boundary or region is an undesirable off-normal event in magnetic fusion experiments, but an ubiquitous process of cosmological importance in astrophysical plasmas. Parallel transport theory of ambipolar-constrained tail electron loss is known to predict rapid cooling of the parallel electron temperature $T_{e\parallel },$ although $T_{e\parallel }$ is difficult to diagnose in actual experiments. Instead direct experimental measurements can readily track the perpendicular electron temperature $T_{e\perp }$ via electron cyclotron emission. The physics underlying the observed fast drop in $T_{e\perp }$ requires a resolution. Here two collisionless mechanisms, dilutional cooling by infalling cold electrons and wave-particle interaction by two families of whistler instabilities, are shown to enable fast $T_{e\perp }$ cooling that closely tracks the mostly collisionless crash of $T_{e\parallel }.$ These findings motivate both experimental validation and reexamination of a broad class of plasma cooling problems in laboratory, space, and astrophysical settings.

Magnetic confinement of a fusion-grade plasma in the Laboratory has shown that extreme care must be exercised in the design of the magnetic fields in order to sustain a nearly collisionless plasma^1,2^. In space and astrophysical systems, the low particle density and extremely large spatial scale can easily accommodate nearly collisionless plasmas^3–11^. Large-scale cooling of such nearly collisionless plasmas, especially in the presence of structure formations in a tenuous astrophysical plasma background, becomes a plasma transport process of significant cosmological importance^8–11^. One of the most interesting features is the so-called cooling flow, the presence of which defies the normal transport closure^8,9,12,13^ such as the collisional Braginskii^14^ and collisionless flux-limiting^15^ forms of electron thermal conduction, but is allowed if the plasma kinetics is fully accounted for^16^. Interestingly, a wholly undesirable phenomenon in the magnetic confinement experiment, the so-called thermal quench (TQ) in the first phase of a tokamak disruption^17,18^, provides a laboratory platform to understand the intriguing *plasma kinetics* underlying the rapid cooling of a nearly collisionless plasma against a cooling boundary, which can be the chamber wall or injected high-Z pellets^19,20^.

The millisecond and sub-millisecond time-scale TQ^21–23^ of a magnetically confined plasma is thought to be dominated by plasma parallel transport, especially electron thermal conduction, along open (stochastic) magnetic field lines. The most extreme and astrophysically relevant regime has a magnetic connection length $L_B$ comparable to or even significantly shorter than the core plasma mean-free-path $\lambda _{mfp}.$ The conventional wisdom is that in such low collisionality regime, the electron parallel conduction flux would follow the so-called flux-limiting (FL) form, $q_{en} \sim n_e T_{e\parallel } v_{th,e\parallel },$ with $v_{th,e\parallel }=\sqrt{ k_B T_{e\parallel }/m_e}$ the local parallel electron thermal speed^15,24^. As a result, the TQ time duration would follow the scaling $\tau _{TQ}^{FL} \propto m_e^{1/2} (n_0\ln \Lambda )^{-1/4}L_B^{3/4}T_0^{0}$ with $n_0$ and $T_0$ the initial plasma density and temperature, respectively, and $\ln \Lambda$ the Coulomb logarithm (detailed derivations of $\tau _{TQ}^{FL}$ and the following $\tau _{TQ}^\parallel$ can be found in the method section), but the cooling flow can not be supported. Recent simulations and analysis^16,25^ showed instead that ambipolar transport constrains the electron parallel thermal conduction so that the cooling flow is supported, and $\tau _{TQ}^\parallel \propto m_i^{3/4}m_e^{-1/4}\left( n_0 \ln \Lambda \right) ^{-1/4} L_B^{3/4} T_0^0.$ Remarkably, a recent analysis of EAST disruption experiments^26^ revealed an extremely weak dependence of $\tau _{TQ}$ on $T_0,$ $\tau _{TQ}^{\perp , exp} \propto T_0^{-0.08},$ consistent with the predicted scaling with $T_0$ ($\tau _{TQ}^\parallel \propto T_0^0$), notwithstanding that the critical $m_i/m_e$ scaling in $\tau _{TQ}^\parallel$ in relation to $\tau _{TQ}^{FL},$ as well as the presence/absence of a cooling flow, remain to be checked by experiments.

The apparent agreement in $T_e$ scaling of $\tau _{TQ}$ actually belies a critical physics gap between theory and experimental measurements, in that parallel thermal conduction in a nearly collisionless plasma cools $T_{e\parallel }$^16,25,27–29^ but the electron cyclotron emission (ECE) diagnostics^22,23,26^ measure $T_{e\perp }.$ The conventional wisdom is that the *collisional* cooling of $T_{e\perp }$ can be fast once $T_{e\parallel }$ becomes sufficiently low, since collisional electron temperature isotropization follows $\partial T_{e\perp }/\partial t = - \left( T_{e\perp } - T_{e\parallel }\right) /\tau _{e\perp }^c$ with $\tau _{e\perp }^c \propto m_e^{1/2}\left( n_e \ln \Lambda \right) ^{-1} T_{e\parallel }^{3/2}$^30,31^. Indeed, the collisional cooling ($\tau _{e\perp }^c$) of $T_{e\perp }$ would be mostly independent of the initial core temperature $T_0$ and can be quite fast if $T_{e\parallel }$ becomes sufficiently low. In this scenario, the TQ is separated into two distinct phases, with durations $\tau _{TQ}^\parallel$ and $\tau _{TQ}^\perp =\tau _{e\perp }^c,$ both of which have no or weak dependence on $T_0,$ but with very different scalings with respect to $L_B$ ($\tau _{TQ}^\parallel \propto L_B^{3/4}$ v.s. $\tau _{TQ}^\perp \propto L_B^0$) and plasma density ($\tau _{TQ}^\parallel \propto n_0^{-1/4}$ v.s. $\tau _{TQ}^\perp \propto n_0^{-1}$). How short $\tau _{TQ}^\perp$ can be is mostly set by how low $T_{e\parallel }$ can be cooled in the first phase.

This paper describes the physics of *collisionless* cooling of $T_{e\perp },$ which produces qualitatively different TQ history in that there is no longer a separate phase for $T_{e\perp }$ as $T_{e\parallel ,\perp }$ now follow the same $\tau _{TQ}$ scaling previously given for $T_{e\parallel },$*i.e.*$\tau _{TQ}\approx \tau _{TQ}^\parallel \propto m_i^{3/4} m_e^{-1/4} n_0^{-1/4} L_B^{3/4} T_0^0.$ Our analysis indicates that this is an unavoidable scenario as long as $L_B \le \lambda _{mfp}$ at the onset of TQ and plasma Debye length $\lambda _D$ is much shorter than the system length $L_B.$ There are actually two distinct mechanisms that can contribute to the collisionless cooling of $T_{e\perp }.$ The first is the infalling cold electrons from the cooling zone which has a much lower temperature $T_w \ll T_0.$ Here “infalling” refers to the fact that these cold electrons follow the ambipolar electric field into the core plasma, where they reduce the core $T_{e\perp }$ by dilutional cooling.^16,32^ The second mechanism is the result of electron temperature isotropization via wave-particle interaction, by self-excited electromagnetic waves in the whistler range. There are actually two distinct types of whistler instabilities involved. What gets excited first is the trapped-electron whistler (TEW) mode, previously identified in^29^, but is now significantly modified by the infalling cold electrons. This first type of whistler instabilities drives the truncated electron distribution towards a bi-Maxwellian with $T_{e\perp }\gg T_{e\parallel }.$ The ensuing, second type of whistler instability is the well-known temperature anisotropy driven whistler (TAW) mode^33,34^, which can aggressively bring down $T_{e\perp }.$ Upon the nonlinear saturation of the whistler instabilities, $T_{e\perp }/T_{e\parallel }$ approaches the marginality of TAW, which depends on the plasma beta. As a general guidance, for modest cooling ($T_0/T_{e\parallel } \lesssim 10$), dilutional cooling is sufficient to align $T_{e\perp }$ cooling with that of $T_{e\parallel }.$ Deep cooling ($T_0/T_{e\parallel } \gtrsim 10^2$) critically relies on the two types of whistler instabilities to work in sequence. In combination, these two distinct collisionless mechanisms align the $T_{e\perp }$ cooling with the rapid $T_{e\parallel }$ quench in a nearly collisionless plasma, from the modest to deep cooling regimes. The requirement of $L_B \gg \lambda _D,$ which is certainly satisfied in laboratory magnetic confinement experiments, ensures that the whistler dynamics is fast in relaxing the electron temperature anisotropy compared with $T_{e\parallel }$ cooling. The laboratory experiments on tokamak and stellarator thermal quench thus offer an exciting opportunity to validate these interesting kinetic physics. The new physics findings reported here would also motivate a reexamination of this class of plasma cooling problems in space and astrophysical settings, where nearly collisionless plasmas are more commonly observed.

## Results

To elucidate the physics of collisionless $T_{e\perp }$ cooling, we first briefly review how $T_{e\parallel }$ is rapidly cooled by parallel transport. In a nearly collisionless plasma, $T_{e\parallel }$ cooling is the result of tail electron loss^16^, which produces a truncated distribution function in $v_\parallel$ that has the cutoff speed $v_c = \sqrt{2e \Delta \Phi _{RF}/m_e}$ so $f_e(v_\parallel > v_c)=0.$ Here the reflecting potential $\Delta \Phi _{RF}$ arises in order to enforce ambipolar transport, and a decreasing $\Delta \Phi _{RF}$ leads to $T_{e\parallel }$ cooling. For deep cooling, i.e. $T_{e\parallel } \ll T_0,$ the reflecting potential satisfies $v_c \ll v_{th,e}\equiv \sqrt{k_BT_0/m_e}.$ In the middle of the open magnetic field line of connection length $L_B,$ the electrostatically trapped electron distribution of the core plasma can thus be modeled as

1 $$\begin{aligned} f_t(v_\parallel ,v_\perp )=\frac{2{(1-\alpha )} n_e}{erf (v_c/v_t)\sqrt{\pi }v_t^3}e^{-(v_\parallel ^2+v_\perp ^2)/v_t^2}\Theta (1-v_\parallel ^2/v_c^2), \end{aligned}$$

where $v_t=\sqrt{2}v_{th,e}$ for simplicity of expressions, $n_e$ is the electron density, $1-\alpha$ is the density fraction of the trapped electrons, and $erf (x)$ and $\Theta (x)$ are the error and Heaviside step function, respectively.

$T_{e\perp }$***cooling by dilution.*** In a nearly collisionless plasma, the cold electrons near the cooling boundary, where electron energy is taken out by impurity radiation and/or wall recycling, will move upstream into the core plasma by following the ambipolar electric field, gaining the kinetic energy of $e \Delta \Phi _{RF}.$ These infalling cold electrons can be modeled as

2 $$\begin{aligned} f_r^\pm =\frac{{\alpha } n_e}{v_{w}^2v_c}e^{-v_\perp ^2/v_{w}^2}\delta (1\pm v_\parallel /v_c), \end{aligned}$$

where $v_{w}=\sqrt{2T_w/m_e}$ and $\delta (x)$ is the delta-function. Here “+” and “−” represent the infalling electron beam from the right and left boundary, respectively. With the defined trapped and infalling electron distributions, we obtain the total electron distribution

3 $$\begin{aligned} f_e=f_t + f_r^++f_r^-. \end{aligned}$$

In fact, $\alpha$ denotes the fraction of the cold electron beam density. During TQ, one can assume that $f_e$ at $v_\parallel =0$ doesn’t change^16^, so $n_e/erf (v_c/v_t)\times (1-\alpha )=n_0.$ This implies $\alpha \le \alpha _{max}=1-erf (v_c/v_t)$ since $n_e\le n_0.$ One then finds that the infalling cold electrons would dilutionally cool the core $T_{e\perp }$ to $\alpha T_{w} + (1-\alpha ) T_0,$ with the constraint of $\alpha \le \alpha _{max}$ as noted earlier.

***Modification of trapped electron whistler (TEW) instability by infalling cold electron beams.*** The truncated electron distribution $f_t$ of Eq. (1) is known to drive robust whistler instabilities^29^. To understand the impact of the infalling cold electron population $f_r^\pm$ of Eq. (2), we substitute $f_e$ of Eq. (3) into the dispersion of whistler wave along the magnetic field with normal mode $\exp \left( ik x- i\omega t\right) ,$^35^

4 $$\begin{aligned}0&=1-\frac{k^2c^2}{\omega ^2}+\nonumber \\&\quad\frac{\omega _{pe}^2}{n_e\omega }\int \int \left[ \left( 1-\frac{kv_\parallel }{\omega }\right) \frac{\partial f_e}{\partial v_\perp ^2}+\frac{kv_\parallel }{\omega }\frac{\partial f_e}{\partial v_\parallel ^2}\right] \frac{v_\perp ^3 dv_\perp dv_\parallel }{\omega -kv_\parallel -\omega _{ce}}, \end{aligned}$$

where we have ignored the effect of ions assuming $\omega _{ci}\ll \omega <\omega _{ce}$ with $\omega _{ce,i}$ the electron/ion gyro-frequency, and $\omega _{pe}$ is the plasma frequency. This leads to the dispersion relation

5 $$\begin{aligned} D(\omega ,k)=1-\frac{k^2c^2}{\omega ^2}+(1-\alpha )D_t+\alpha D_r=0, \end{aligned}$$

where

6 $$\begin{aligned} D_t&=\frac{\omega _{pe}^2}{erf (\hat{v}_c)\sqrt{\pi }\omega ^2}\left[ \frac{\omega }{kv_t}\int _{-\hat{v}_c}^{\hat{v}_c}\frac{e^{-\hat{v}_\parallel ^2}}{\hat{v}_\parallel -\xi }d\hat{v}_\parallel +\frac{\hat{v}_ce^{-\hat{v}_c^2}}{\hat{v}_c^2-\xi ^2}\right] ,\end{aligned}$$

7 $$\begin{aligned} D_r&=-\frac{\omega _{pe}^2}{\omega ^2}\left[ \frac{\hat{v}_c^2-\omega \xi /(kv_t)}{(\hat{v}_c+\xi )(\hat{v}_c-\xi )} +\frac{(\xi ^2+\hat{v}_c^2)\hat{v}_{w}^2}{2[(\hat{v}_c+\xi )(\hat{v}_c-\xi )]^2} \right] , \end{aligned}$$

with $$\hat{v}_{\parallel , c, w}=v_{\parallel , c,w}/v_t,$$ and $$\xi =(\omega -\omega _{ce})/kv_t$$.

The contribution of the infalling cold electron beams can be examined by setting $\alpha =1.$ Important insights can be readily obtained in the limiting cases of $\hat{v}_c\ll 1$ and $\hat{v}_c\gg 1.$ In the former case, both $\xi + \hat{v}_c$ and $\xi -\hat{ v}_c$ approximate to $i\gamma /(kv_t)$ and thus contribute equally to $D_r,$ where $i\gamma /(kv_t)>\hat{v}_c$ with $\xi \approx 0$. Let’s further assume that $\Vert \omega \xi /(kv_t)\Vert \ll \hat{v}_{w}^2,$ in the limit of $k^2c^2\gg \omega ^2$ we obtain

8 $$\begin{aligned} \gamma =\frac{v_{w}}{\sqrt{2} c} \omega _{pe}. \end{aligned}$$

Notice that the growth rate in Eq. (8) is similar to that of TEW^29^ with $v_{w}$ replacing $v_{t}$ as the free energy for the instability. This is not surprising since both $f_t$ and $f_r^{\pm }$ are like delta-function in $v_\parallel$ for whistler modes in the limit of $v_c\ll \omega /k\sim v_t.$ On the other hand, for large $v_c,$ only one of $\hat{v}_c\pm \xi$ satisfies the resonant condition, so

9 $$\begin{aligned} \gamma =\frac{v_{w}}{2c}\omega _{pe}, \end{aligned}$$

for $k^2c^2\gg \omega _r^2.$ The different factors in Eqs. (8, 9) results from the different number of resonant conditions. In contrast to the TEW instability^29^ for $v_c\gg v_t,$ where the growth rate $\gamma \propto \exp (-\hat{v}_c^2/2),$ significantly decreases with increasing $\hat{v}_c,$ $\gamma$ in Eq. (9) is independent of $\hat{v}_c.$ In reality, the decompressional cooling of $T_{e\parallel }^{beam}$ for the infalling cold electrons will produce a lower $T_{e\parallel }^{beam}<T_w$ compared with the $T_{e\parallel }^{beam}=T_w,$ but not $T_{e\parallel }^{beam}=0$ as represented by $\delta (1\pm v_\parallel /v_c)$ in Eq. (2). The $\gamma$s in Eqs. (8,9) are thus upper bounds for a quantitative estimate.

Comparing Eqs. (8, 9) with the growth rates of the pure TEW mode ($\alpha =0$) in^29^, we find that the impact of the infalling cold electrons for small but finite $\alpha$ on TEW instability depends on $\hat{v}_c.$ For small $\hat{v}_c\ll 1,$ we have

10 $$\begin{aligned} \gamma = R \frac{v_t}{\sqrt{2} c} \omega _{pe}, \end{aligned}$$

with $R\equiv \sqrt{(1-\alpha )+\alpha \hat{v}_{w}^2},$ so the cold beams with $\hat{v}_{w}\ll 1$ will reduce the growth rate of TEW since $R<1$ for $\alpha >0.$ Specifically, for $\alpha =\alpha _{max}\approx 1-2\hat{v}_c/\sqrt{\pi }$ with $\hat{v}_c\ll 1,$ we have a reduced factor of $R\approx \sqrt{2\hat{v}_c/\sqrt{\pi }+ \hat{v}_{w}^2}.$ Whereas, for $\hat{v}_c\gg 1,$ the impact of cold electron beams depends on $\alpha$. In the TQ, $\alpha$ is small with $\alpha <\alpha _{max}\approx \exp (-\hat{v}_c^2)/(\sqrt{\pi }\hat{v}_c)$ for $\hat{v}_c\gg 1.$ As such, the imaginary part of *D*, excluding the factor $\omega _{pe}^2/\omega ^2,$ satisfies

11 $$\begin{aligned} \frac{\omega _r}{(kv_c)^2} \gamma - \frac{kv_te^{-\hat{v}_c^2}}{2\sqrt{\pi } \gamma } +\alpha \frac{\omega _{ce}}{2\gamma }=0. \end{aligned}$$

It follows that the infalling cold electrons of a tiny fraction in the TQ will weaken the whistler instability through the third term in Eq. (11). For a general finite $\alpha$ in other scenarios, the growth rate is determined by the real part of *D*, 

12 $$\begin{aligned} \gamma =\left[ 1-(1-\alpha )\frac{\omega _r\omega _{pe}^2}{k^3v_cc^2}\right] ^{-1/2}\frac{v_{w}}{2 c} \omega _{pe}, \end{aligned}$$

which is the same as Eq. (9) for $\alpha = 1.$ Since the factor in the bracket is larger than unity for $\alpha <1,$ the growth rate in Eq. (12) is smaller than that in Eq. (9) for $\alpha =1$ but will be larger than that for $\alpha =0.$

**Fig. 1:**
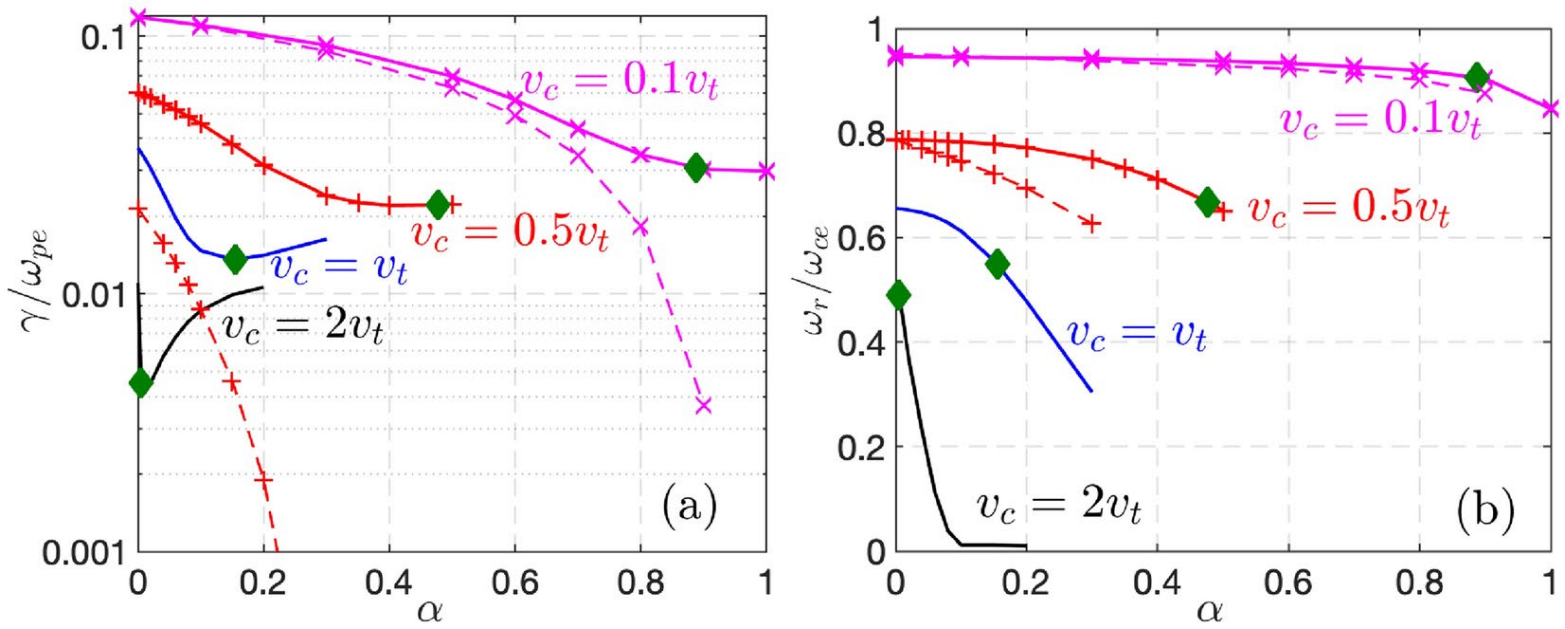
Growth rate (**a**) and frequency (**b**) of the most unstable mode from Eq. (5) are shown in solid lines for $$\omega _{ce}=\omega _{pe},$$$$c=5v_t,$$$$v_{w}=0.3v_t$$ ($$T_w\approx 0.09 T_0$$) and $$\beta _0\equiv 8\pi n_eT_0/B_0^2=4\%,$$ where the parameters correspond to a TQ of plasma with $$T_0=10keV$$^16^. For $$n_e\approx 10^{20}m^{-3},$$$$B_0\approx 3.2 T.$$ The diamonds label the upper limit of $$\alpha =\alpha _{max}$$ in the TQ process. For small $$v_c =0.5 v_t$$ (marked with “+” markers) and $$0.1v_t$$ (marked with “$$\times$$” markers)., growth rates and frequencies for the *equivalent* TAW instability are shown in dashed lines. For $$v_c\ge v_t,$$*equivalent* TAW is stable and not shown.

Figure 1 shows the numerical solutions of Eq. (5) for $v_{w}=0.3v_t$ ($T_w=0.09T_0$) but different $v_c$ and $\alpha,$ which agree well with Eqs. (10-12). We also plot the results from a bi-Maxwellian with equivalent perpendicular and parallel temperatures, defined as

13 $$\begin{aligned} \frac{T_{e\perp }}{T_0}=R^2,~\frac{T_{e\parallel }}{T_0}=(1-\alpha )\left[ 1-\frac{2\hat{v}_c\exp (-\hat{v}_c^2)}{\sqrt{\pi }erf (\hat{v}_c)}\right] + 2\alpha \hat{v}_c^2. \end{aligned}$$

It shows that the infalling cold electrons will also stabilize the *equivalent* TAW instability, mainly through the reduction of the temperature anisotropy for $\hat{v}_c> \hat{v}_w*\sqrt{1/2-\hat{v}_c\exp (-\hat{v}_c^2)/(\sqrt{\pi }erf (\hat{v}_c))},$ which is readily satisfied for $\hat{v}_w\ll 1.$ More importantly, it shows that trapped electrons provide a more robust drive for the whistler instability than temperature anisotropy even in the presence of infalling cold electrons, so that the former will excite the whistler waves first in a TQ. Another interesting and important finding is that the growth rate of the most unstable mode will increase with decreasing $v_c$ (due to the cooling of $T_{e\parallel }$ in the TQ) despite the infalling cold electrons (e.g., see the upper bounds of $\alpha$ marked by the diamonds). Therefore, the whistler instabilities and the associated wave-particle interactions will be greatly enhanced with the cooling of $T_{e\parallel }.$

Comparing with the TAW dispersion analysis in the method section, one can see that the growth rate and frequency of the most unstable mode without infalling electrons are nearly the same for TEW with small $v_c\ll v_t$ and the equivalent TAW with large temperature anisotropy, both of which would be far above marginality in these limits. Closer to marginality, TEW and TAW are known to have drastically different dependences on the plasma beta. Specifically, the TAW excitation requires the plasma beta, defined with $T_{e\parallel }$ in the form of $\beta _{e\parallel }=8\pi n_eT_{e\parallel }/B_0^2,$ to surpass a threshold value^34,36^ but TEW does not.^29^

**Fig. 2:**
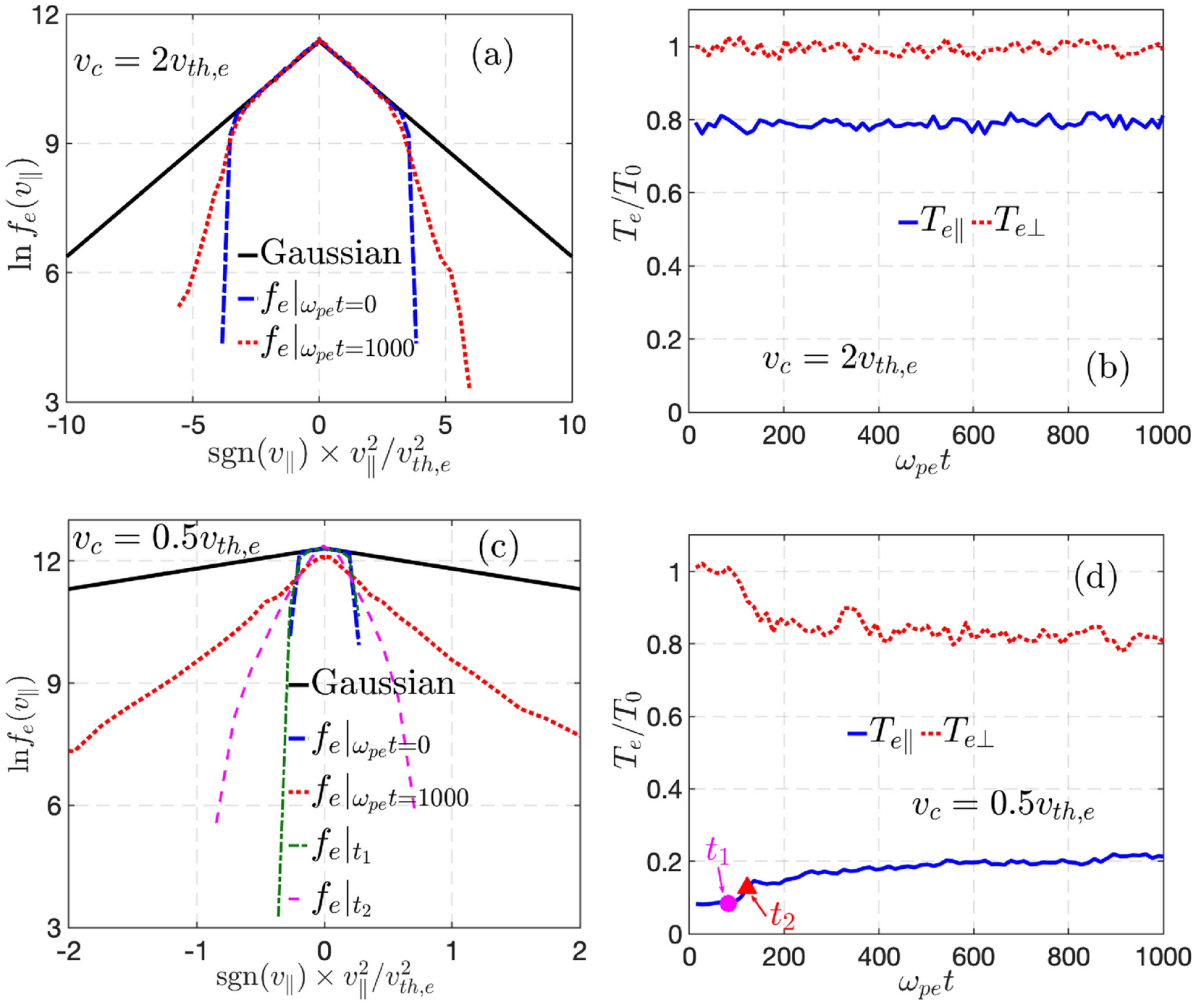
$f_e(v_\parallel )$ and $T_{e\parallel ,\perp }$ at plasma center are shown for the cases of $v_c=2v_{th,e}$ (**a**,**b**) and $0.5v_{th,e}$ (**c**,**d**) from periodic box simulations without infalling cold electrons (similar results exist for simulations with infalling cold electrons). The plasma parameters are the same as Fig. 1. For $v_c=0.5v_{th,e}$, $f_e$ and $T_e$ at $t_{1}=81\omega _{pe}^{-1}$ and $t_{2}=108\omega _{pe}^{-1}$ are shown.

***Two-stage process of***$T_{e\perp }$***cooling by two kinds of whistler instabilities in sequence.*** In the thermal quench of nearly collisionless plasmas dominated by tail electron loss along the magnetic field, the TEW instability, despite the stabilizing effect of infalling cold electrons, will be excited first, for its much higher growth rate. Interestingly, because the primary drive for this mode is the sharp cut-off of the distribution at the electrostatic trapping boundary $v_\parallel =v_c,$ it saturates quickly with a modest amount of smearing of the trapped-passing boundary^37^. Consequently, there is a rather limited amount of $T_{e\perp }$ cooling if $v_c > v_{th,e}.$ To illustrate this physics, we perform VPIC^38^ simulations in a periodic box with initially truncated electron distribution given in Eq. (1). Figure 2a,b shows the result for $v_c=2 v_{th,e},$ a case with unstable TEW mode but no corresponding *equivalent* TAW instability (see Fig. 1). Nonlinear saturation of the TEW modes produces a smeared cut-off boundary but no appreciable temperature isotropization.

For deep cooling of $T_{e\parallel },$ which corresponds to $v_c$ significantly smaller than $v_{th,e},$ collisionless cooling of $T_{e\perp }$ takes a two-stage route, which is shown in Fig. 2c,d for the case of $v_c=0.5 v_{th,e}.$ The quick saturation of TEW from $t_1$ to $t_2$ produces an approximate bi-Maxwellian with $T_{e\parallel } \ll T_{e\perp }$ around $t_2.$ This should be followed by the excitation of TAW^33,34^ as expected from the numerical solution of the dispersion relation in Fig. 1, the saturation of which produces further temperature isotropization after $t_2.$ The eventual residual temperature anisotropy ($A_r\equiv T_{e\perp }/T_{e\parallel }$) that this two-stage collisionless cooling of $T_{e\perp }$ can reach, is set by the marginality condition for TAW^34,36^.

Whether one can observe a clearly delineated two-stage process as described above depends on the separation in the growth rates of the most unstable TEW and TAW modes. Here we give two cases to elucidate two important factors that can affect this separation. The first factor is that the growth rate of TEW has a sensitive dependence on the inevitable relaxation of the trapped-passing boundary at $v_c.$ The first case is shown in Fig. 2 which has $\beta _0=4\%$ and $v_c=0.5v_{th,e}.$ The growth rates of the most unstable TEW and TAW modes are $\gamma _{TEW }\approx 0.07\omega _{pe}$ and $\gamma _{TAW }\approx 0.05\omega _{pe},$ respectively, which should be distinguishable if the VPIC resolution is high enough. However, as noted in Ref.^37^, the distribution in VPIC cannot sustain a cutoff Maxwellian with a discontinuity and the trapped-passing boundary will be slightly smoothed as shown in Fig. 2 starting from the first time step of the simulations. As a result, the growth rate of TEW from VPIC simulations should be smaller than that of an exact cutoff Maxwellian^37^, providing $\gamma _{TEW }^{VPIC }\approx 0.05\omega _{pe}$ by VPIC. This makes the separation of TEW and TAW instabilities by growth rates in the linear stage nearly impossible, although the relaxation of the electron distribution shown in Fig. 2 is still indicative of a two-stage process that is consistent with the different ways of TEW and TAW reaching their marginalities in nonlinear saturation.

**Fig. 3:**
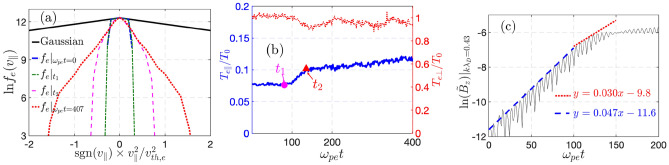
$f_e(v_\parallel )$ (**a**), $T_{e\parallel ,\perp }$ (**b**), and perturbed $B_z$ in logarithmic scale at $k\lambda _D\approx 0.43$ and its fitting (**c**). Here $t_1=81\omega _{pe}^{-1}$ and $t_2=135\omega _{pe}^{-1}.$ The simulation setup is the same as Fig. 2(**c**,**d**) but with doubled $\omega _{ce}$ and hence $\beta _0=1\%$.

The second factor of interest is that the TAW mode, in contrast to TEW, has a strong dependence on plasma beta. For the second case, we perform another VPIC simulation with the same plasma parameters except for a doubled $\omega _{ce}$ and hence lower plasma beta $\beta _0=1\%,$ for which $\gamma _{TEW }^{VPIC }=0.05\omega _{pe}$ is nearly unchanged but $\gamma _{TAW }$ is reduced to $\gamma _{TAW }\approx 0.03\omega _{pe}.$ The characteristics of $f_e(v_\parallel )$ and $T_e$ are the same as Fig. 2c,d, while the perturbed $B_z$ for the most unstable mode near $k\lambda _D\approx 0.43$ is shown in Fig. 3. From the fitted growth rates, one can clearly observe the TEW and TAW instabilities at the early and late phases of the linear instability stage, where the growth rates agree well with the expected values. The relaxation of the distribution at $t_1, t_2,$ and later time in Figs. 3 and 2 shows the same characteristics with TEW causing a smoothing of the trapped-passing boundary at $v_c$ while TAW producing strong thermalization in $v_\parallel$ and subsequent isotropization of $T_{e\parallel }$ and $T_{e\perp }.$

**Fig. 4:**
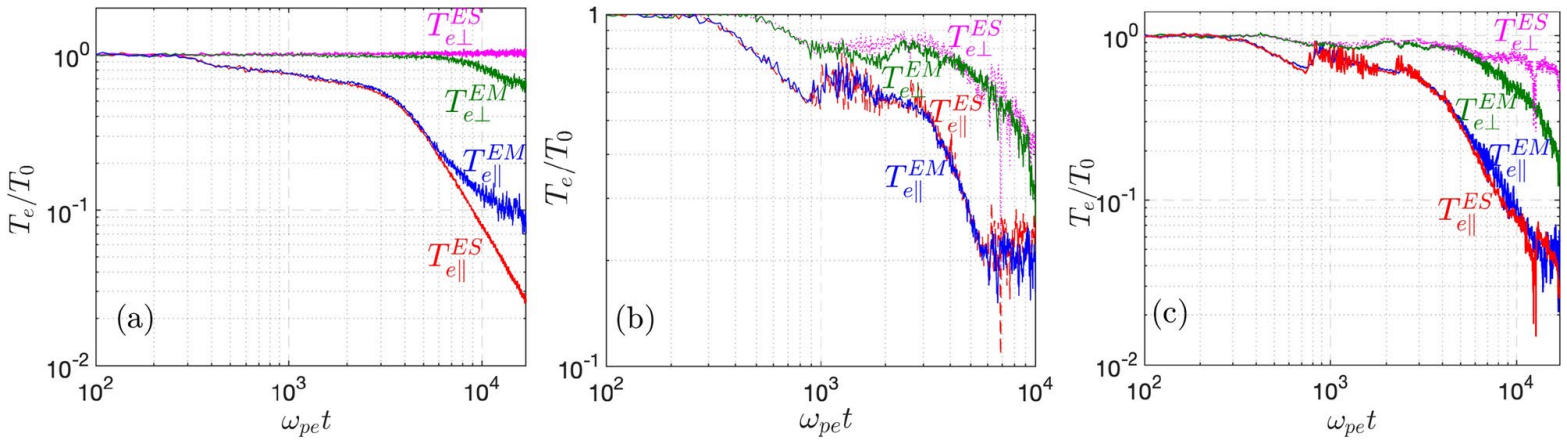
$T_{e\parallel ,\perp }(t)$ at the center of plasmas from both electromagnetic (EM, with whistler modes) and electrostatic (ES, without whistler modes) TQ simulations, where the simulation setup is shown in the method section.** a** Is for the absorbing boundary,** b** and** c** are for plasma recycling boundary with $T_w=0.1T_0$ and $T_w=0.01T_0$, respectively. A reduced domain of $L_x=2L_B=1400\lambda _D$ and ion mass $m_i=100m_e$ are employed so that $\tau _{TQ}\equiv L_B/c_s \sim 10^4\omega _{pe}^{-1}$ for the ion front stage. Actual tokamak plasma of much longer $L_B$ has $\tau _{TQ}$ scaling up as $\propto L_B^{3/4}.$.

***Relative importance of different collisionless***$T_{e\perp }$***cooling mechanisms from fully kinetic TQ simulations.*** In an integrated TQ simulation over open magnetic field lines with connection length $L_B,$ the dynamical cooling of both $T_{e\parallel }$ and $T_{e\perp }$ is self-consistently accounted for. Since fully kinetic VPIC^38^ simulations would need to resolve the Debye length $\lambda _D,$ we will explore down-scaled simulations that retain the extreme low collisionality of the physical system ($L_B/\lambda _{mfp} \ll 1$) but shrink the simulation domain to $L_B/\lambda _D\sim 10^3.$ Previous comparison between theoretical analysis and simulation results^25^ has shown that such down-scaled simulations capture the $T_{e\parallel }$ cooling physics accurately for $L_B/\lambda _{mfp} \sim 0.05.$ Specifically, the TQ is controlled by four propagating fronts that are originated from the cooling boundary^16^: two of them are fast electron fronts with speeds at $\sim v_{th,e}$ that lead to fast but only moderate cooling of $T_{e\parallel }$ ($T_{e\parallel }\tilde{>}0.6T_0$ with large $v_c$), while the other two are slow ion fronts with speeds at ion sound speed $\sim c_s$, which control the deep cooling of $T_{e\parallel }$ via continuously reducing $v_c$. Notice that the aforementioned cooling flow is formed between the ion fronts as a result of the largely reduced electron thermal conduction from the flux-limiting formula due to ambipolar transport constraint. Thus, the rate of $T_{e\parallel }$ cooling and hence $v_c$ reduction is $\sim v_{th,e}/L_B$ at the fast electron fronts phase and $\sim c_s/L_B$ at the slow ion fronts phase^25^. In reality, these rates are much lower than $\omega _{pe}$ by 5-6 orders of magnitude due to the large $L_B$, leading to a large time-scale separation between $T_{e\parallel }$ cooling and $(T_{e\perp },T_{e\parallel })$ isotropization by whistler instabilities. For the latter,the most unstable TEW/TAW mode, for appreciable $T_{e\parallel }$ cooling of $T_{e\parallel }\lesssim 0.3T_0$ (i.e., $v_c\lesssim v_t$, where the wave-particle interaction begins to cool $T_{e\perp }$ as shown later in Fig. 4) in fusion plasma with $\beta _0\sim 1\%,$ has wavenumber $k_m\sim \lambda _D^{-1}$ and growth rate $\gamma _m\gtrsim 10^{-2}\omega _{pe}$ from our analysis and thus their nonlinear saturations would occur at $\sim 10^2 \omega _{pe}^{-1}$. Such large time-scale separation allows the $T_{e\perp }$ cooling physics to be reliably established in the down-scaled simulations as well, which, as reported here, can resolve the most active whistler modes in space and time over the slower process of $T_{e\parallel }$ cooling as long as $L_B/\lambda _D \gtrsim 10^3$ is satisfied. An extra complication is that the TEW mode is particularly sensitive to collisional damping^37^ so down-scaled simulations should have $\lambda _{mfp} > 10^6\lambda _D,$ so $L_B/\lambda _{mfp} < 10^{-3},$ which can be easily accommodated in down-scaled collisionless simulations.

To isolate the different cooling mechanisms in the integrated $(T_{e\parallel }, T_{e\perp })$ cooling simulations using VPIC, we contrast electromagnetic (EM) with electrostatic (ES) simulations. The former can retain the whistler instabilities, while the latter can not. Using a plasma absorbing boundary to remove the possibility of dilutional cooling, simulations in Fig. 4a demonstrate that the whistler modes are self-excited in the EM simulation to produce collisionless $T_{e\perp }$ cooling. With a plasma recycling boundary condition to mimic a cooling boundary in a TQ that radiatively clamps the plasma temperature to $T_w \ll T_0$^16,25^, simulations in Fig. 4b,c now retain the dilutional cooling mechanism as seen in the $T_{e\perp }$ cooling in the ES simulations, while the $T_{e\perp }$ cooling due to the whistler instabilities can be extracted from the difference of $T_{e\perp }$ in the ES and EM simulations. Fig. 4b shows that the dilutional cooling of $T_{e\perp }$ can dominate over that of the whistler modes for a modest amount of cooling with $T_w/T_0=0.1$, while if deep cooling is needed as shown in Fig. 4c for $T_w=0.01T_0$, the dilutional cooling (in the ES curve) is rather ineffective. As such, the whistler instabilities retained in the EM simulation are now essential in the collisionless cooling of $T_{e\perp }$ that closely tracks the cooling history of $T_{e\parallel }$ (notice that the slope of the temperature cooling history in the log-log scale provides the scaling of the TQ duration).

**Fig. 5:**
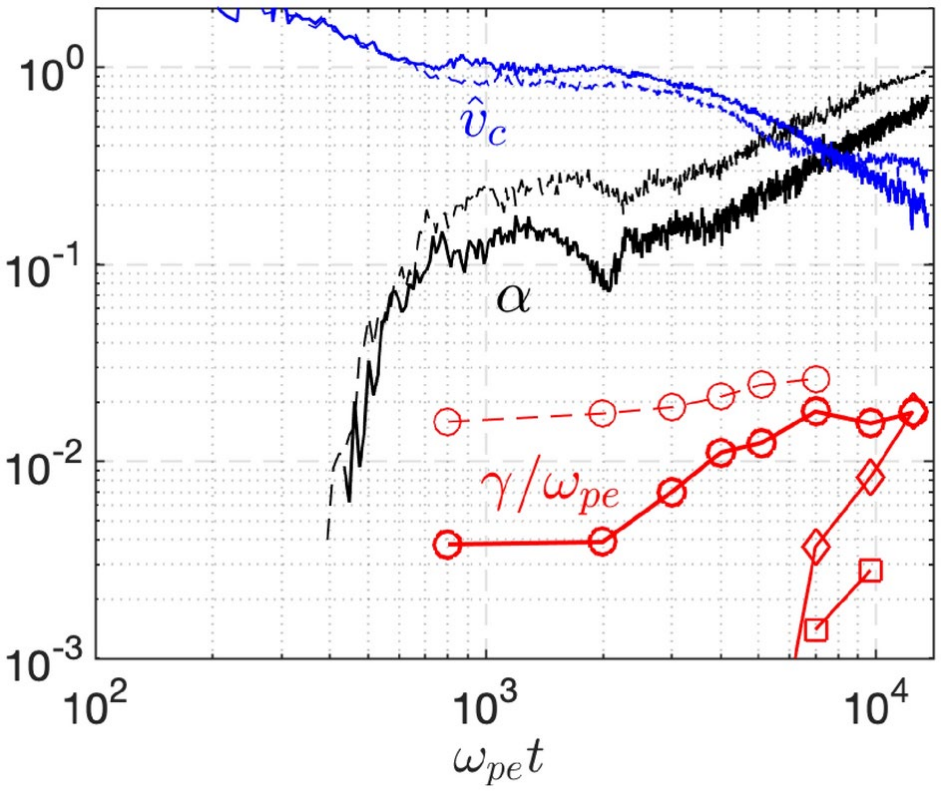
Infalling cold electron fraction $\alpha$ and the cutoff velocity (computed from Eq. (13)) at the plasma center from the EM simulation in Fig. 4b for $T_w=0.1T_0$ (dashed lines) and Fig. 4c for $T_w=0.01T_0$ (solid lines). From these $\alpha$ and $\hat{v}_c$, growth rates of the most unstable TEW mode are computed at a few moments (circles) starting from the modest cooling of $T_{e\parallel }=0.6-0.7T_0$. The *equivalent* TAW is calculated as well at the same moments, which is only unstable at a later time for $T_w=0.01T_0$ (diamonds) but always stable for $T_w=0.1T_0$ (not shown). The growth rates of actual TAW using $T_{e\perp }$ from the $T_w=0.01T_0$ simulation (squares) are much lower at a late time because marginality is being approached.

Along with Figs. 4, 5 brings together the key points that were introduced earlier in isolation. Deep cooling of $T_{e\parallel }$ comes from the drop of $v_c/v_t$ to $\sim 0.1,$ during which the infalling cold electrons take on an increasingly larger fraction $\alpha \rightarrow 1.$ While the TEW instability is robustly unstable from an early time $\omega _{pe}t\lesssim 6000$, deep cooling of $T_{e\perp }$ for $T_w=0.01T_0$ at $\omega _{pe}t\gtrsim 6000$ requires the excitation of strong TAW modes in a two-stage process. The residual $A_r$ is set by the marginality of TAW. Specifically, the marginal $A_r$ is a decreasing function^34,36^ of $\beta _{e\parallel }$, i.e., $A_r=1+ S_e/\beta _{e\parallel }^{\zeta }$ with $\zeta \sim 0.5$ and $S_e\sim 0.5$ for $0.1\lesssim \beta _{e\parallel }\lesssim 1$. For smaller $\beta _{e\parallel }\sim 1\%$ in magnetic fusion plasmas, which is further decreasing in the TQ process, the parameter $\zeta$ is even smaller and following^34,36^, linear analysis of the TAW instability for $\gamma = 10^{-3}\omega _{pe}$ suggest that $\zeta \approx 0.41$ and $S_e\approx 0.37$ for $4\times 10^{-5}\lesssim \beta _{e\parallel }\lesssim 2\times 10^{-3}$ where $0.001\lesssim \beta _{e\perp }\equiv A_r\beta _{e\parallel }\lesssim 0.01$. As such, the saturated temperature anisotropy will increase in the TQ with $T_{e\parallel }$ cooling but remain at a reasonable value $A_r\lesssim 10$ for $T_{e\parallel }$ cooling from 10 *keV* to $\sim 10s~eV$.

We shall point out that for such small $\beta _{e\perp }$, the plasma kinetic energy is nearly conserved during the temperature isotropization process, i.e., $2T_{e\perp }+T_{e\parallel } \approx const.$. For a plasma with strong temperature anisotropy satisfying $A\equiv T_{e\perp }/T_{e\parallel }\gg A_r \gg 1$, $T_{e\perp }$ is slightly changed with a fraction of $\sim 1/A_r$, while $T_{e\parallel }$ is significantly increased from $T_{e\perp }/A$ to $T_{e\perp }/A_r$. Therefore, the more natural way to express the bound of the temperature anisotropy can utilize $\beta _{e\perp }$ rather than $\beta _{e\parallel }$ so that one can easily predict the saturated plasma state. Mathematically, $A_r\approx S_e/\beta _{e\parallel }^\zeta \approx S_eA_r^\zeta /\beta _{e\perp }^\zeta$ such that $A_r\approx S_e^{1-\zeta }/\beta _{e\perp }^{\zeta /(1-\zeta )}$.

## Conclusions

To conclude, we note that thermal quench experiments of the Laboratory magnetic fusion plasmas offer a rare opportunity to study the kinetic transport physics underlying the conductive cooling of nearly collisionless plasmas more commonly found in space and astrophysical settings. There is a compelling case, from theory and simulation, that collisionless cooling will bring down $T_{e\perp }$ proportionally to follow a crashing $T_{e\parallel }.$ The specific mechanisms involve dilutional cooling by infalling cold electrons, and wave-particle interaction through a two-stage process driven by two kinds of whistler wave instabilities. Particularly, the trapped electrons, as a result of the ambipolar transport, provide a robust drive to the whistler instability that can be damped by the infalling electrons. The large time-scale separation between the TQ and $(T_{e\perp }, T_{e\parallel })$ isotropization by whistler instabilities enables decoupled simulations for the latter with fixed $v_c$ in the periodic box and downscaled simulations for the integrated TQ dynamics. Both have revealed that the saturation of the whistler modes and the associated temperature isotropization depend strongly on the effective temperature anisotropy, where the residual temperature anisotropy for deep cooling of $T_{e\parallel }$ is near the marginality of the TAW instability.

It should also be noted that realistic plasmas, especially the astrophysical ones, can have significant gradients of magnetic field and gravitational forces, both of which can significantly impact the collisionless charged particle motion. These can introduce further complications to electron parallel transport. For example, the mirror force associated with magnetic field strength modulation is known to produce exotic parallel thermal conduction that has parallel heat flux (component) flowing in the opposite direction of the temperature gradients^39^. Accounting for their roles in the thermal quench of a nearly collisionless plasma would be of considerable interest and importance.

## Methods

**Fig. 6:**
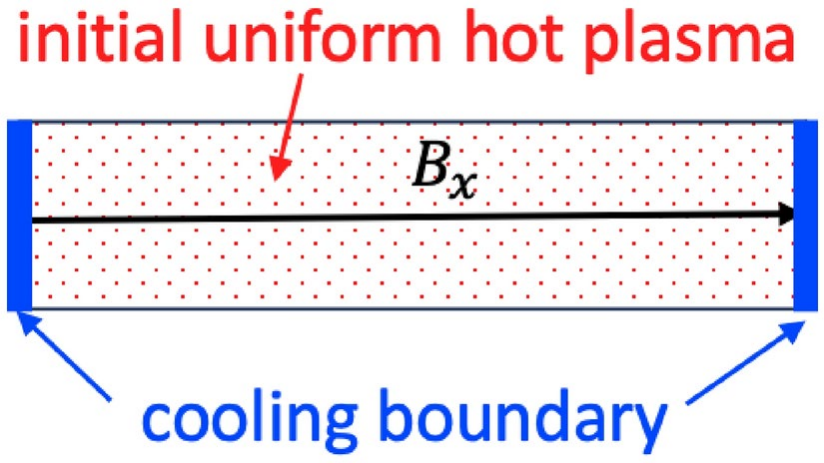
Schematic view of the 1D (along *x* direction) thermal quench simulation setup. The cooling boundary is considered for two distinct cases, thermobath and absorbing boundaries as described in the Methods, to contrast for physics insights. The hot plasma mean-free-path is long compared to the domain size.

We deployed fully kinetic one-dimensional-three-velocity (1D3V) down-scaled simulations using the VPIC^38^ code, in both the electromagnetic and electrostatic versions, to investigate the thermal quench of a nearly collisionless plasma in the presence of a cooling spot, where the parallel transport physics will dominate. The simplest problem setup to decipher the parallel cooling physics is to unwind the open field lines into a one-dimensional slab with length $$L_x=1400\lambda _D,$$ e.g., see Fig. 6. For the thermal quench simulations in Fig. 3b,c, cooling spots are introduced at both the boundaries as thermobaths (which is also called recycling boundary), that re-inject electron-ion pairs equal to the ions across the boundary with a clamped temperature of $$T_w\ll T_0$$ (we vary $$T_w=0.1T_0$$ and $$T_w=0.01T_0$$ in the simulations). As the cooling front forms and propagates into the plasma from the cooling boundary,^16^ some of the recycled” cold electrons can follow the ambipolar electric field and penetrate through the cooling front toward the plasma center. These are the infalling cold electrons. To isolate the dilutional cooling and the cooling due to the wave-particle interaction, for Fig. 3a we have introduced the absorbing boundaries that absorb all the particles reaching the boundary. The initial condition is a magnetized plasma with $$n_e=10^{20}m^{-3},$$$$T_0=10keV$$ (in a Maxwellian distribution) and magnetic field with $$B_0 =3.2T.$$ For the decoupled simulations for the whistler instability shown in Fig. 2, all the setup is the same as the thermal quench case except that the boundary condition is periodic and the initial distribution of electrons is a cut-off Maxwellian in the parallel direction and Maxwellian in the perpendicular directions. To quantify the fraction of the infalling cold electrons in the core of the plasma, we labeled the recycled electrons as a new species. In all the simulations, the grid size in the simulations is chosen as $$dx=0.1\lambda _D,$$ which resolves the Debye length of the cold plasma near the boundary. The markers per cell are 5000 to have a low level of noise. We used reduced ion mass $$m_i=100m_e$$ to speed up the thermal quench simulations.

The plasma density, flow, and temperature are computed directly from the plasma distribution *f* in the following way

14 $$\begin{aligned} n=&\int f d^3v, \end{aligned}$$

15 $$\begin{aligned} nV_\parallel =&\int v_\parallel f d^3v, \end{aligned}$$

16 $$\begin{aligned} nT_\parallel =&m\int (v_\parallel -V_\parallel )^2f d^3v, \end{aligned}$$

17 $$\begin{aligned} nT_\perp =&m\int v_\perp ^2 fd^3v. \end{aligned}$$

**Derivations of **$\tau _{TQ}^{FL}$**and**$\tau _{TQ}^{\parallel }$. For both the flux-limiting case $q_{en} \propto n_e T_{e\parallel }^{3/2} m_e^{-1/2}$ and the ambipolar-constrained case $q_{en} \propto n_e T_{e\parallel }^{3/2} m_i^{-1/2}$, the evolution of center $T_{e\parallel }$ follows

18 $$\begin{aligned} n_e\frac{\partial T_{e\parallel }}{\partial t} \approx -\frac{\partial q_{en}}{\partial x}\approx \frac{q_{en}}{L_B}, \end{aligned}$$

which gives

19 $$\begin{aligned} \frac{T_{e\parallel }}{T_0} \propto \left( \delta + v_{th,0}^{FL,\parallel }t/L_B\right) ^{-2}, \end{aligned}$$

where $v_{th,0}^{FL}=\sqrt{T_0/m_e}$ and $v_{th,0}^{\parallel }=\sqrt{T_0/m_i},$ respectively, and numeric factor $\delta \sim 1$. As the drop of $T_{e\parallel },$ there will be a transition from collisionless to collisional cooling. Following Ref.^25^, we assume that such a transition would occur when $q_{en}$ transits from the collisionless formula to the Braginskii form with $q_e\propto n_eT_e^{3/2}K_nm_e^{-1/2},$ where the Knudsen number $K_n=\lambda _{mfp}/L_B\propto T_e^2/(L_B n_e\ln \Lambda )$ with $\ln \Lambda$ the Coulomb logarithm. As a result, the transition temperature $T_r$ is simply given by

20 $$\begin{aligned} \frac{T_r^{FL}}{T_0}\propto K_{n,0}^{-1/2},~\frac{T_r^\parallel }{T_0}\propto K_{n,0}^{-1/2}(m_e/m_i)^{1/4}. \end{aligned}$$

Then ignoring $\delta$ in Eq. (19) for deep cooling of $T_{e\parallel }$ from $T_0$ to $T_r,$ we obtained

21 $$\begin{aligned} \tau _{TQ}^{FL} \propto m_e^{1/2} (n_0\ln \Lambda )^{-1/4}L_B^{3/4}T_0^{0}, \end{aligned}$$

and

22 $$\begin{aligned} \tau _{TQ}^\parallel \propto m_i^{3/4}m_e^{-1/4}\left( n_0 \ln \Lambda \right) ^{-1/4} L_B^{3/4} T_0^0. \end{aligned}$$

**TAW instability for strong temperature anisotropy.** For a bi-Maxwellian, the dispersion relation for TAW takes the simple form

23 $$\begin{aligned} 1-\frac{k^2c^2}{\omega ^2}+\frac{\omega _{pe}^2}{\omega ^2}\left[ A+\left( \frac{\omega }{kv_{t\parallel }}+A\zeta _e\right) Z(\zeta _e)\right] =0, \end{aligned}$$

where $A=T_{e\perp }/T_{e\parallel }-1,$ $v_{t\parallel } = \sqrt{2T_{e\parallel }/2},$*Z*(*x*) is the plasma dispersion function, and $\zeta _e=(\omega -\omega _{ce})/kv_{t\parallel }.$ For strong temperature anisotropy $A\gg 1,$ we assume $\Vert \zeta _e\Vert \approx \gamma /kv_{t\parallel } \gg 1$ with $\omega _r\approx \omega _{ce}$ due to the small $v_{t\parallel }.$ As a result,

24 $$\begin{aligned} A+\left( \frac{\omega }{kv_{t\parallel }}+A\zeta _e\right) Z(\zeta _e) \approx i\frac{\omega _r}{\gamma } + \frac{Ak^2v_{t\parallel }^2}{2\gamma ^2}\left( 1+2i\frac{\omega _r-\omega _{ce}}{\gamma }\right) . \end{aligned}$$

For $k^2c^2\gg \omega ^2,$ which is the case for plasmas with large $\beta _0\gtrsim 1\%,$ the real part of the dispersion relation then gives

25 $$\begin{aligned} \gamma =\frac{v_{t\perp }}{\sqrt{2}c}\omega _{pe}, \end{aligned}$$

with $v_{t\perp }=\sqrt{2T_{e\perp }/m_e}=\sqrt{A}v_{t\parallel }$. While the imaginary part of Eq. (24) gives

26 $$\begin{aligned} \frac{\omega _r}{\omega _{ce}}=1-\frac{\omega _{pe}^2}{2k^2c^2} = 1-\frac{v_{t\perp }^2/c^2}{2k^2\lambda _{D\perp }^2}, \end{aligned}$$

where $$\lambda _{D\perp }=v_{t\perp }/\omega _{pe}$$.

## Data Availability

Source data is provided in this paper. Additional data that support the findings of this study are available from the corresponding authors upon reasonable request. The code that supports the plots within this paper is available from the corresponding authors upon reasonable request.
